# 

**Published:** 1908-06-13

**Authors:** 


THE MODERN MEDICAL JOURNAL.

Telephone Number: 2734 Gerrard.

NEW SERIES. No. 65, Vol. III.

No. 1137. Vol. XLIV.

SATURDAY, JUNE 13TH, 1908.

PRICE THREEPENCE.

